# Intra-cardiac pressure drop and flow distribution of bicuspid aortic valve disease in preserved ejection fraction

**DOI:** 10.3389/fcvm.2022.903277

**Published:** 2022-08-24

**Authors:** Shirin Aliabadi, Alireza Sojoudi, Murad F. Bandali, Michael S. Bristow, Carmen Lydell, Paul W. M. Fedak, James A. White, Julio Garcia

**Affiliations:** ^1^Department of Biomedical Engineering, University of Calgary, Calgary, AB, Canada; ^2^Stephenson Cardiac Imaging Centre, Libin Cardiovascular Institute, Calgary, AB, Canada; ^3^Circle Cardiovascular Imaging, Advanced Technologies, Calgary, AB, Canada; ^4^Department of Cardiac Sciences, Cumming School of Medicine, University of Calgary, Calgary, AB, Canada; ^5^Libin Cardiovascular Institute, Calgary, AB, Canada; ^6^Department of Radiology, University of Calgary, Calgary, AB, Canada; ^7^Alberta Children’s Hospital Research Institute, University of Calgary, Calgary, AB, Canada

**Keywords:** 4D-flow MRI, regurgitation, flow components, pressure drop, left ventricle, bicuspid aortic valve, diastolic function

## Abstract

**Background:**

Bicuspid aortic valve (BAV) is more than a congenital defect since it is accompanied by several secondary complications that intensify induced impairments. Hence, BAV patients need lifelong evaluations to prevent severe clinical sequelae. We applied 4D-flow magnetic resonance imaging (MRI) for in detail visualization and quantification of *in vivo* blood flow to verify the reliability of the left ventricular (LV) flow components and pressure drops in the silent BAV subjects with mild regurgitation and preserved ejection fraction (pEF).

**Materials and methods:**

A total of 51 BAV patients with mild regurgitation and 24 healthy controls were recruited to undergo routine cardiac MRI followed by 4D-flow MRI using 3T MRI scanners. A dedicated 4D-flow module was utilized to pre-process and then analyze the LV flow components (direct flow, retained inflow, delayed ejection, and residual volume) and left-sided [left atrium (LA) and LV] local pressure drop. To elucidate significant diastolic dysfunction in our population, transmitral early and late diastolic 4D flow peak velocity (E-wave and A-wave, respectively), as well as E/A ratio variable, were acquired.

**Results:**

The significant means differences of each LV flow component (global measurement) were not observed between the two groups (*p* > 0.05). In terms of pressure analysis (local measurement), maximum and mean as well as pressure at E-wave and A-wave timepoints at the mitral valve (MV) plane were significantly different between BAV and control groups (*p*: 0.005, *p*: 0.02, and *p*: 0.04 and *p*: <0.001; respectively). Furthermore, maximum pressure and pressure difference at the A-wave timepoint at left ventricle mid and left ventricle apex planes were significant. Although we could not find any correlation between LV diastolic function and flow components, Low but statistically significant correlations were observed with local pressure at LA mid, MV and LV apex planes at E-wave timepoint (*R*: −0.324, *p*: 0.005, *R*: −0.327, *p*: 0.004, and *R*: −0.306, *p*: 0.008, respectively).

**Conclusion:**

In BAV patients with pEF, flow components analysis is not sensitive to differentiate BAV patients with mild regurgitation and healthy control because flow components and EF are global parameters. Inversely, pressure (local measurement) can be a more reliable biomarker to reveal the early stage of diastolic dysfunction.

## Introduction

The beating heart’s ultimate function is to continuously and efficiently pump blood to supply body organs constantly. Many cardiac abnormalities can perturb normal intricate intra-cardiac hemodynamics flow patterns and local pressure, triggering higher energy dissipation and pumping deficiency (1, 2). Bicuspid aortic valve (BAV), as the most common congenital valvular disease, is not an exception (3). This abnormal development of the aortic valve (AV) during early fetal life can be the leading cause of subsequent complex disorders in different age groups (4, 5). It has become increasingly evident that congenital BAV, cusp fusion morphology and secondary abnormalities, including BAV-induced regurgitation alter blood flow patterns in the environments connected to the valve (6–8). Chronic aortic regurgitation (AR) with the severity of moderate to severe is associated with increased left ventricle (LV) workload, LV diastolic dysfunction and remodeling, cardiac fibers deformability and finally, imminent heart failure (HF) (9, 10). However, hemodynamic alteration and its effects on the LV chamber due to asymptomatic AR with mild severity and normal systolic function are scant in the literature.

Approaches for intra-cardiac left ventricular (LV) four flow components including direct flow (DF), retained inflow (RI), delayed ejection (DE), and residual volume (RV) under healthy and abnormal conditions, have been proposed using 4-dimensional time-resolved blood flow velocities (4D-flow) (11, 12). Multidimensional LV flow components have been characterized under healthy condition by measurable parameters for quality and effective preparation for ejection by Eriksson et al. (13). Any alteration in these flow characteristics by BAV-induced regurgitation may prove useful as subclinical markers of LV impairment. Nevertheless, the role of LV flow components in BAV subgroups as one of the imaging biomarkers in clinical decision-making needs scrutiny aligned with continuous advancements in medical imaging modalities.

Furthermore, pressure gradient has been emphasized as another important biomarker for evaluating the severity of cardiovascular and valvular deficiencies (14, 15). To determine the *in vivo* pressure gradients in clinical practice, diagnostic catheterization is used as the gold standard. Although this method is reliable with a low risk (16), it is invasive and associated with various complications such as potential side effects and ionizing radiation dose. Hence, this technique is not recommended as a suitable choice for regular follow-up (17) and routine diagnostic pressure assessment (18). Alternatively, maximum velocity is derived non-invasively from standard clinical ultrasound (US), and it is converted to pressure gradient by the simplified Bernoulli equation [Δ*p* = 4 V^2^_max_] (19, 20). Nonetheless, assumption dependency, limited acoustic window, operator dependency, and variability in velocity assessment resulting from beam alignment are the main shortcoming of the US clinical tool (21, 22).

According to recent literature, obtained pressure mapping based on time-resolved three-dimensional (3D) phase-contrast magnetic resonance imaging (so-called 4D-flow MRI), shows an excellent agreement to pressure drop acquired from heart catheterization (23, 24). Lately, 4D-flow MRI, among various diagnostic techniques, has massively enhanced the understanding of multifaceted and pulsatile blood flow patterns within the heart and mediastinal vessels by uncovering and characterizing different flow parameters and advanced flow biomarkers (25, 26). 4D flow MRI also offers a more robust and precise computation of temporally and spatially distributed pressure drop within the complex hemodynamic conditions of the region of interest with fewer assumptions than the other methods (24, 27, 28). However, MRI and the recently derived 4D flow technique have limitations that prevent them as the first line of diagnosing, ranging from pricy and cases with contraindication for MRI (patients with implanted pacemakers or defibrillator) to long scan time related to 4D flow MRI. In recent years, some innovative imaging techniques have been proposed to accelerate electrocardiogram (ECG) gated multidimensional phase-contrast data (4D flow MRI) over several cardiac cycles and to make it a clinically applicable approach (29).

Integration of morphological data and 3D cine phase-contrast cardiovascular magnetic resonance (PC-CMR) flow data has allowed addressing unresolved physiological and clinical questions of BAV mediated hemodynamics on the surrounding environment such as LV and thoracic aorta clinically (30–32). However, BAV-induced regurgitation impairment on the left ventricle as a hardworking cardiac chamber during the cardiac cycle needs additional attention (33). In this study, we aimed: (I) to assess the left ventricular flow components (Global measurement), (II) to analyze left-sided pressure drop (local measurement) during the cardiac cycle by applying 4D flow MRI for differentiating BAV cohorts with mild regurgitation and healthy control. Therefore, we hypothesize that both intracardiac flow components and pressure drop can be reliable 4D flow MRI parameters to identify abnormal left ventricular workflow in BAV patients with mild regurgitation and preserved ejection fraction (pEF).

## Methods and materials

### Study cohort

For this study, we retrospectively identified *n*: 51 BAV patients with mild regurgitation (age: 39.8 ± 11.5, female: 18) and *n*: 24 healthy controls (age: 38.4 ± 14.0, female: 8) with negligible mean age difference (*p* = 0.65). A minimum sample size of 11 subjects per cohort was estimated based on Engineering’s sample size estimation (34). Patients were recruited as a pre-defined sub-study of our local observational clinical cardiovascular registry. The study was coordinated by commercial software (cardioDI™, Cohesic Inc., Calgary, AB, Canada) for the routine capture of patient informed consent, health questionnaires and for standardized collection of MRI-related variables. Patients were distinguished by standardized coding of clinical referral indications for BAV, including BAV morphology characterization. Exclusion criteria for patients included the history of myocardial infarction, non-ischemic cardiomyopathy, complex congenital heart disease, MRI-coded moderate-severe mitral valve (MV) insufficiency, or significant systolic dysfunction [left ventricle ejection fraction (LVEF) < 50%]. Healthy volunteers ≥17 years of age were recruited and underwent identical workflow and were required to have no known cardiovascular disease, hypertension or diabetes and have no contraindications for MRI (35).

All subjects provided written informed consent. The Institutional Review Board (IRB) approved this study at our institution. All research procedures were performed in agreement with the Declaration of Helsinki.

### Cardiac magnetic resonance data acquisition

All subjects underwent a standardized imaging protocol consistently using 3T MRI scanners (Skyra, Prisma, Siemens, Erlangen, Germany). Multi-planar segmented ECG gated, time-resolved balanced steady-state free precession (SSFP) cine imaging in 4-chamber, 3-chamber, 2-chamber, and short-axis views were achieved covering the whole heart at end-expiration for functional assessment of LV. Moreover, for volumetric assessment, 3D magnetic resonance angiography (MRA) of the whole heart was acquired by administrating 0.2 mmol/kg gadolinium contrast (Gadovist, Bayer, Mississauga, ON, Canada). Then, whole-heart 4D flow MRI was performed 5–10 min following contrast administration, using free-breathing retrospective ECG-gated for comprehensive intracardiac 3D *in vivo* volumetric blood flow assessment. The parameters were set as follows: velocity encoding range in all direction (venc): 150–200 cm/s, flip angle: 15°, spatial resolution: 2.0–3.6 mm × 2.0–3.0 mm × 2.5–3.5 mm, temporal resolution: 25–35 ms, phases: 30, bandwidth: 455–495 Hz/Pixel, echo time: 2.01–2.35 ms, pulse repetition time: 4.53–5.07 ms. The overall scan times varied between 8 and 12 min, depending on the physiologically based factors, defined scan parameters and respiratory gating efficiency.

### Cardiac magnetic resonance imaging and 4D-flow analysis

**FIGURE 1:**
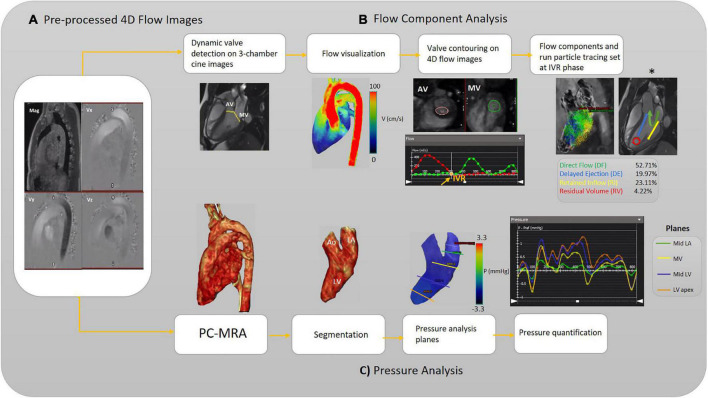
Workflow illustration. **(A)** Pre-processing of the raw 4D-flow data. **(B)** Analyzing flow components. The different left ventricular (LV) blood flow components are shown schematically (asterisk). **(C)** Analyzing pressure, including calculation of 3D PC-MRA from the magnitude and 3D velocities to extract the left side of the heart for each subject (3D segmentation). The pressure-time curve is achieved to measure pressure at each pre-defined plane and desired timepoint. Aortic valve (AV), mitral valve (MV), isovolumetric relaxation (IVR), left ventricle (LV), Mid (middle part), left atrium (LA), and phase-contrast magnetic resonance angiography (PC-MRA).

**FIGURE 2:**
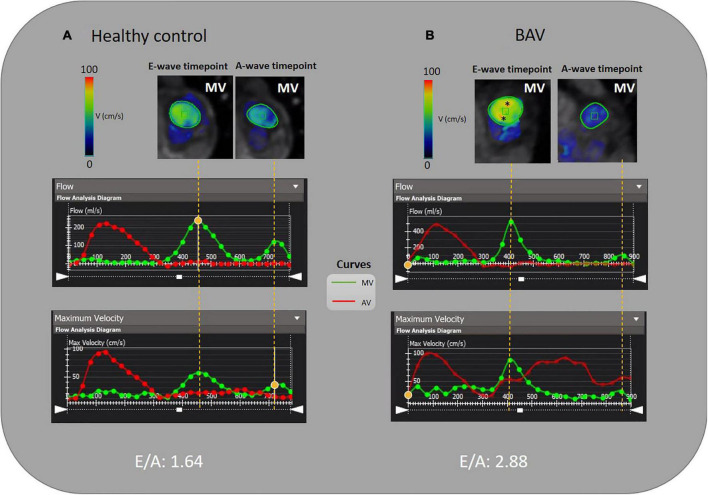
E/A measurement by 4D flow MRI analysis. **(A)** A 46-year-old healthy female and **(B)** A 35-year-old BAV patient male with mild regurgitation. MV is contoured semiautomatically and dynamic MV plane was set throughout a cardiac cycle. The acquired flow-time diagram is turned into a peak velocity-time diagram to measure E and A-wave peak velocity and E/A. Asterisks (*) indicate increased velocity at E-wave timepoint in BAV with mild regurgitation compared to the healthy subject.

Standard clinical reading was performed using cvi42 v5.11.5 (Circle Cardiovascular Imaging Inc., Calgary, AB, Canada). We derived functional parameters of LV from cardiac MR images. Left ventricular end-systolic volume (LVESV) and left ventricular end-diastolic volume (LVEDV) obtained and indexed to body surface area (BSA). The cvi42 v5.11.5 4D-flow module was used to analyze the left ventricular flow components (DF, DE, RI, and RV) and left-sided [left atrium (LA) and LV] pressure drop during a cardiac cycle. Moreover, to elucidate significant diastolic dysfunction in the pEF, transmitral early and late diastolic 4D flow peak velocity (E-wave and A-wave, respectively), as well as E/A ratio variable, were acquired. The schematic workflows are provided in Figures 1, 2, including the pre-processing steps and 4D-flow analysis. AV and MV regurgitation ranges were classified in accordance with available guidelines (36). The acquisitions underwent pre-processing to correct for Maxwell terms, eddy current-induced phase offset, and velocity aliasing within the software environment (Figure 1A). Afterward, each subject underwent LV flow components, pressure and E/A ratio analyses as follows:

#### Flow components analysis

Intracavitary LV blood flow was divided into four components over the cardiac cycle, namely: (I) DF, the blood volume that enters and leaves the LV during the same cardiac cycle; (II) RI, blood volume that enters the LV during the diastolic phase and does not eject in systolic phase in the same cardiac cycle; (III) DE flow, blood volume that retained into the LV and ejected in the next systole and; (IV) RV, the amount of blood that remains in LV for two cardiac cycles or more.

The left ventricular flow components analysis starts with selecting the long-axis three-chamber SSFP cine image preferably to automatically localize static AV and MV planes on each frame (Figure 1B). An automatic valve tracking machine learning algorithm that has been authenticated as a consistent 4D flow-derived blood flow measurement was initially applied to recognize each valve area (AV and MV) based on each frame’s long-axis three-chamber cine image (37). In the three-chamber cine views, tissue feature tracking was applied to turn the static planes to dynamic according to valves motion during the cardiac cycle. Color-coded flow visualization was used for in detail semiautomatic locating and contouring of AV and MV, and our single analyst modified valves’ locations when it was necessary for precise contouring on 4-D flow images throughout the cardiac cycle. The isovolumetric relaxation (IVR) phase was set where both aortic and MVs flow profiles were minimized (both valves were closed) at the end of the systolic phase. The four components of the left ventricular flow (DF, DE, RI, and RV) were acquired according to a previously accredited method (11, 13). In this regard, pathline particles were emitted from each voxel within the LV chamber and separated into four flow components. In addition, aortic and MVs flow measurements were provided in terms of total volume, peak velocity, and regurgitant fraction (Figure 1B).

#### Pressure analysis

Rendered phase-contrast magnetic resonance angiography (PC-MRA) acquisitions of the whole heart were used for LA and LV 3D segmentation. Then, five static planes, including a reference plane, were accurately positioned orthogonal to the segmented LA and LV longitudinal axis, and contoured manually in succession at the segmented left side of the heart as follows: upper part of the LA (reference plane), LA middle part, MV, LV middle part, LV apex. For each plane, the maximum and average pressure and pressure at four cardiac time points were measured, including peak systole, end-systole at the time of IVR, early and late diastolic transmitral pressure (E-wave and A-wave, respectively). The four cardiac time points locations were determined using acquired flow-time curves for each assigned plane to measure pressure according to the pressure-time diagram (Figure 1C). The dedicated analysis software (cvi42) provides relative pressure fields from velocity MRI data by solving the pressure Poisson’s equation considering the complex geometry of the cardiovascular system (38).

#### E/A acquisition

Although cardiac catheterization is the gold standard method and echocardiography has been suggested as a reliable imaging technique to assess diastolic function, we have employed 4D flow MRI capability to measure this parameter. Early and late transmitral diastolic peak velocity (E-wave and A-wave, respectively) were measured by dynamic mitral plane obtained by MV contouring throughout all phases of the cardiac cycle in the ventricle flow components analysis by 4D flow MRI. E-wave and A-wave timepoints were obtained by the mitral flow-time curve, and then flow-time curve turned into a peak velocity-time curve to measure the maximum velocities at E-wave and A-wave timepoints. Consequently, we attained the E/A variable to uncover any significant diastolic dysfunction as per available normal and abnormal clinically used E/A ranges. In this study, 4D flow MRI-derived mitral filling patterns have been graded as follows: E/A ≤ 1 as impaired relaxation, 1 < E/A < 2 as normal/pseudonormal, and 2 ≤ E/A as restrictive filling (Figure 2) (39).

### Statistical analysis

Statistical analyses were conducted using IBM SPSS (Version 27). Initially, the Shapiro–Wilk’s test was used to evaluate the type of distribution, whether they are normally distributed or not. Based on the distribution, unpaired two-tailed equal variance student’s *t*-test and Mann–Whitney *U* test were used to detect any significant difference between BAV and control subjects for flow components, pressure drop and LV diastolic dysfunction. In addition, Pearson’s product-moment correlation coefficient and Spearman’s rank correlation coefficient were used depending on the type of variables. *p*-values less than 0.05 are considered statistically significant.

## Results

### Cohort characteristics

Our study population encompassed a wide age range from 17 to 62 years old, with the prevalence of male sex. Baseline characteristics and the left ventricular functions of BAV and control cohorts have been provided in Table 1. Significant differences in means of all provided parameters, including ejection fraction (EF), cardiac output (CO), and BSA, have not been observed in both healthy controls and BAV cohorts, while left ventricular stroke volume (LVSV) showed significant differences (*p*: 0.02).

**TABLE 1:** Patient demographics and cardiac left-sided function.

Study group			

	Control	BAV	*p*-value
**General parameters**			
*N* (female)	24 (8)	51 (18)	
Age (years)	38.36 ± 14.04	39.77 ± 11.46	0.65
Height (m)	1.71 ± 0.09	1.73 ± 0.12	0.46
Weight (kg)	78.40 ± 25.01	79.75 ± 17.00	0.81
BSA (m^2^)	1.92 ± 0.32	1.95 ± 0.25	0.69
HR (bpm)	63.41 ± 10.87	65.50 ± 10.60	0.50
SBP (mmHg)	112.40 ± 16.84	108.08 ± 14.13	0.41
DBP (mmHg)	69.64 ± 28.74	63.39 ± 11.60	0.29
**Left ventricular function**			
LVEDV (mL)	159.18 ± 45.63	177.52 ± 67.93	0.29
LVEDV index (mL/m^2^)	83.87 ± 18.11	90.67 ± 31.02	0.39
LVEF (%)	61.14 ± 5.27	60.27 ± 8.08	0.67
LVESV (mL)	62.04 ± 21.41	71.63 ± 30.01	0.21
LVESV index (mL/m^2^)	40.44 ± 19.79	36.98 ± 14.82	0.46
LVSV (mL)	76.91 ± 46.74	103.89 ± 46.02	0.02*
LV mass (g)	97.70 ± 34.14	119.74 ± 54.54	0.11
LV mass index (g/m^2^)	48.61 ± 13.85	60.37 ± 23.50	0.05
LV CO (L/min)	6.38 ± 1.45	7.34 ± 2.14	0.09
**Left atrium function**			
LA volume	68.06 ± 18.34	69.51 ± 28.08	0.84
LA volume index	36.97 ± 9.24	35.03 ± 13.31	0.59

BSA, body surface area; HR, heart rate; SBP, systolic blood pressure; DBP, diastolic blood pressure; LVEDV, left ventricular end-diastolic Volume; LVSV, LVEF, left ventricular ejection fraction; LVESV, left ventricular end-systolic Volume; SV, stroke volume; CO, cardiac output, values are shown as mean ± standard deviation or %.

For normally distributed variables unpaired two-tailed equal variance student’s t-test was applied (*Significant for p < 0.05).

For skewed variables Mann–Whitney test for two independent sample was applied (*Significant for p < 0.05).

### Left ventricular flow components

Table 2 shows the means differences of LV flow components (global measurement) in BAV and control groups were not significantly distinguishable (*p* > 0.05). There were negligible associations between LV mass and DE and LV mass with RI (*R*: 0.269, *p*: 0.021 and *R*: 0.269, *p*: 0.022, respectively).

**TABLE 2:** Flow Components.

Parameters	Control	BAV	*p*-value
**Flow components**			
Direct flow (%)	54.13 ± 14.11	54.87 ± 16.71	0.85
Delayed ejection (%)	12.82 ± 6.57	15.44 ± 8.03	0.17
Retained inflow (%)	13.66 ± 7.55	15.13 ± 7.50	0.44
Residual volume (%)	19.38 ± 12.79	14.56 ± 8.94	0.07
**Aortic valve measurements**			
Aortic valve total volume (mL/cycle)	72.90 ± 18.98	80.53 ± 20.88	0.14
Aortic valve peak velocity (cm/s)	114.49 ± 19.84	131.09 ± 57.80	0.17
Aortic valve regurgitation (%)	1.87 ± 1.73	8.27 ± 9.76	0.002*
**Mitral valve measurements**			
Mitral valve total volume (mL/cycle)	65.15 ± 15.28	68.75 ± 19.71	0.44
Mitral valve peak velocity (cm/s)	70.93 ± 18.09	78.83 ± 28.61	0.22
Mitral valve regurgitation (%)	1.20 ± 2.63	3.85 ± 13.41	0.34

Values are shown as mean ± standard deviation or %.

For normally distributed variables unpaired two-tailed equal variance student’s t-test was applied (*Significant for p < 0.05).

For skewed variables, Mann–Whitney test for two independent samples was applied (*Significant for p < 0.05).

### Pressure drop

Planes were positioned, and pressure was remeasured randomly for 25 subjects by the same observer. All data are available in Supplementary Tables 1, 2. According to the Bland-Altman test with upper and lower 95% confidence interval limits for the average difference (Supplementary Table 3), intra-observer variabilities in relative pressure for all planes and timepoints were negligible. Bland-Altman plots are provided for the MV plane at for cardiac timepoints in the Supplementary Figure 1.

**FIGURE 3:**
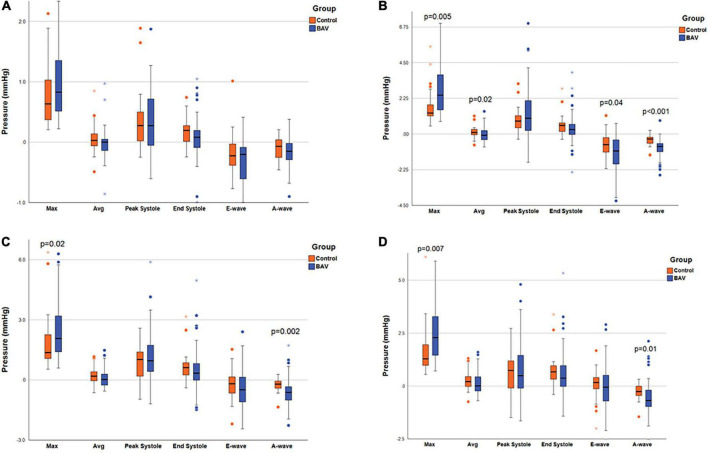
Ventricular pressure measurements. The box plot at the locations of **(A)** LA middle part, **(B)** MV, **(C)** LV middle part, and **(D)** LV apex, pointing out the significant pressure differences between the two groups. Left atrium (LA), mitral valve (MV), and left ventricle (LV). Circles are the outliers and asterisks (*) are the extreme outliers.

The maximum pressure at the MV, LV middle part, and LV apex planes was significantly different between BAV and control groups (*p*: 0.005, *p*: 0.02, and *p*: 0.007, respectively). As such, the pressure differences were significant at the three planes at A-wave timepoints (*p*: <0.001, 0.002, and 0.01, respectively). The average pressure difference, as well as pressure difference at the E-wave timepoint at the MV plane (*p*: 0.02 and *p*: 0.04, respectively), were significant in addition to maximum and A-wave timepoint pressure. Consequently, we could differentiate BAV with mild regurgitation and control groups by measuring local pressure (Table 3 and Figure 3).

**TABLE 3:** Pressure analysis.

Parameters		Control			BAV			*p*-value
			
		Median	Q1	Q3	Median	Q1	Q3	
**Plains pressures (mmHg)**								
Left atrium mid	Max	0.63	0.34	1.07	0.83	0.50	1.43	0.07
	Avg	0.03	−0.06	0.14	0.00	−0.15	0.05	0.10
	Peak systole	0.27	0.02	0.51	0.27	−0.09	0.73	0.84
	End systole	0.19	0.01	0.27	0.08	−0.09	0.20	0.05
	E-wave	−0.23	−0.40	−0.02	−0.20	−0.61	−0.08	0.52
	A-wave	−0.07	−0.26	0.05	−0.15	−0.29	−0.02	0.27
Mitral valve	Max	1.32	1.14	1.87	2.46	1.49	3.88	0.005*
	Avg	0.09	−0.05	0.30	−0.08	−0.37	0.27	0.02*
	Peak systole	0.81	0.39	1.16	0.99	0.21	2.17	0.45
	End systole	0.54	0.12	0.71	0.28	−0.05	0.65	0.11
	E-wave	−0.67	−1.21	−0.21	−1.07	−1.97	−0.35	0.04*
	A-wave	−0.32	−0.64	−0.20	−0.78	−1.13	−0.60	<0.001*
Left ventricle mid	Max	1.36	1.07	2.42	2.07	1.38	3.20	0.02*
	Avg	0.19	−0.04	0.41	0.02	−0.26	0.30	0.07
	Peak systole	1.02	0.18	1.41	0.96	0.39	1.85	0.45
	End systole	0.62	0.20	0.91	0.34	−0.04	0.90	0.14
	E-wave	−0.19	−0.70	0.17	−0.49	−1.17	0.14	0.28
	A-wave	−0.21	−0.42	−0.05	−0.62	−1.02	−0.27	0.002*
Left ventricle apex	Max	1.28	0.96	2.10	2.29	1.43	3.30	0.007*
	Avg	0.21	−0.03	0.45	0.00	−0.25	0.44	0.13
	Peak systole	0.74	−0.10	1.21	0.49	−0.17	1.47	0.96
	End systole	0.67	0.30	1.00	0.38	−0.03	1.01	0.21
	E-wave	0.17	−0.13	0.41	−0.05	−0.70	0.58	0.39
	A-wave	−0.26	−0.47	0.00	−0.68	−0.98	−0.18	0.01*

Max, maximum; Mid, middle part; Avg, average; Q1, the lower or first quartile; Q3, the upper, or third quartile.

Values are shown as median, Q1, and Q3.

For normally distributed variables Unpaired two-tailed equal variance student’s t-test was applied (*Significant for p < 0.05).

For skewed variables, Mann–Whitney test for two independent samples was applied (*Significant for p < 0.05).

**FIGURE 4:**
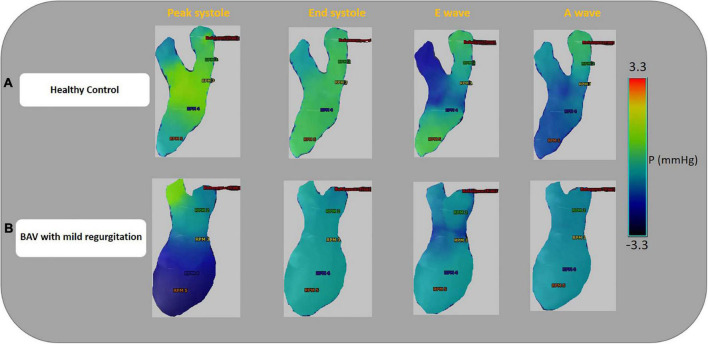
Ventricular pressure examples. Color-coded pressure pattern of the LA and LV throughout four phases of a cardiac cycle (peak systole, end-systole, E-wave, and A-wave). Both subjects have approximately the same flow with pEF. Panel **(A)** shows a 46-year-old healthy female and E/A: 1.58. Panel **(B)** shows a 35.6-year-old BAV patient, male with mild regurgitation and E/A:3.64. The BAV subject shows different local pressure patterns compare to the healthy control. Left atrium (LA), left ventricle (LV), and preserved ejection fraction (pEF).

Left-sided intracardiac local pressure alters under abnormal circumstances. Color-coded pressure shows that pressure at the four cardiac timepoints (peak systole, end-systole, E-wave, and A-wave) in healthy control is different from BAV patients with mild regurgitation severity at the different assigned planes. A BAV patient in grade III (restrictive filling) stage of diastolic dysfunction (E/A > 2) that is associated with reduced LV diastolic compliance, although the ejection fraction is preserved, has been compared with a healthy control in Figure 4. In the systolic phase, the intraventricular pressure is positive in the healthy subject, and the patterns between LA and LV are changed (negative at LV) in the diastolic phase to have a healthy LV filling at early (E-wave) and late (A-wave) diastolic phases. Conversely, the BAV subject shows different local pressure patterns. At the peak-systole pressure tends to be negative in the LV chamber and increase in the diastolic phase indicating LV filling impairment.

We have provided an additional pressure comparison in the four timepoints between a healthy control and a BAV patient in the Supplementary Figure 2.

We found some low correlations between LV function variables and local pressure as follows: between maximum pressure at LA middle part plane and LVCO (*R*: 0.322, *p*: 0.03), pressure at LA middle part at A-wave timepoint and LV mass (*R*: −0.383, *p*: 0.002), pressure at MV plane, at A-wave timepoint and LV mass and LV mass index (*R*: −0.404, *p*: <0.001 and *R*: −0.371, *p*: 0.002). In terms of the association between pressure and LV function variables, pressures at the LV middle part and LV apex at E-wave timepoint have correlations with LV mass and LV mass index. In addition, LVEF did not show any association with the measured local pressures because EF is a global parameter while pressure is a local parameter.

### Diastolic function (E/A ratio)

**FIGURE 5:**
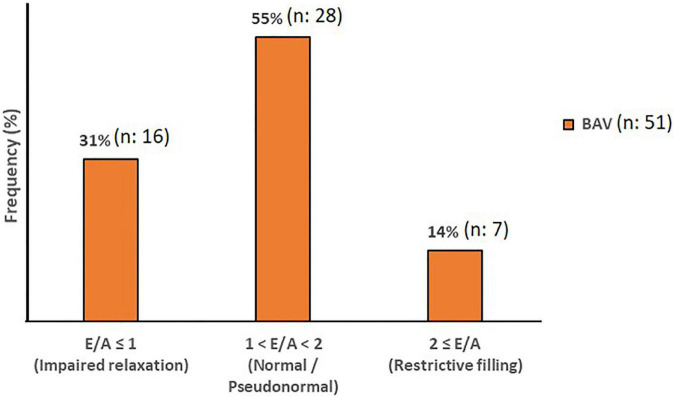
Diastolic Function. The mitral filling patterns categories in BAV with mild regurgitation groups. Normal and Pseudonormal subjects are considered in the same category.

Acquisition of early and late diastolic function ratio by MV contouring using 4D flow MRI analysis software and flow-time and peak velocity-time diagrams indicate abnormal patterns in BAV subjects compared to healthy control. Color-coded velocity of the contoured MV, flow-time and peak velocity-time profile show that BAV subjects are developing diastolic dysfunction even if the systolic function is normal and the regurgitation severity is mild (Figure 5). Nonetheless, accurate diastolic function assessment is too challenging compared to systolic function and other physiological indices such as deceleration time, LA size and pressure, LV compliance, age, sex, heart rate, and so forth need to be considered, especially when it comes to differentiating normal against pseudonormal. Normal and pseudonormal could not be differentiated in our BAV cohort because we did not have access to the additional parameters by 4D flow MRI.

#### Diastolic function and left ventricular flow components

We could not find any correlation between LV flow components and diastolic dysfunction. Correlations just were found between E-wave peak velocity acquired by dynamic plane at MV location in the 4D flow LV analysis with MV total volume and with MV peak velocity (*R*: 0.338, *p*: 0.002 and *R*: 0.512, *p*: <0.001; respectively).

#### Diastolic function and pressure drop

**FIGURE 6:**
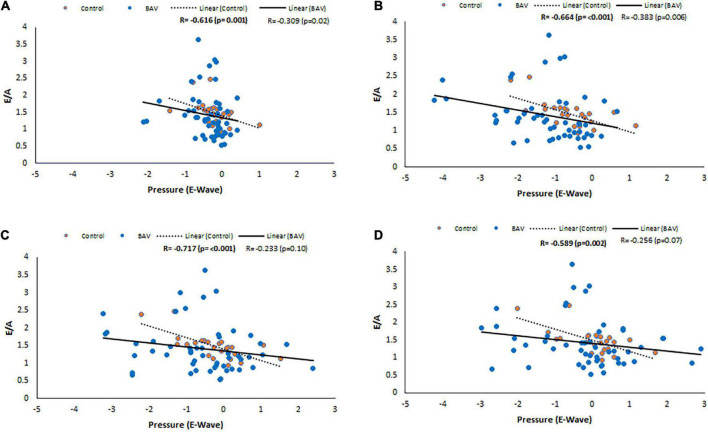
E/A correlations with pressure at E-wave timepoint in control and BAV groups. The locations are shown at **(A)** LA middle part, **(B)** MV, **(C)** LV middle part, and **(D)** LV apex. Left atrium (LA), mitral valve (MV), and left ventricle (LV).

Considering control and BAV cohorts altogether, Diastolic dysfunction (E/A) shows negative and low but statistically significant correlations with pressure at LA middle part, MV, LV middle part and LV apex planes at E-wave timepoint (*R*: −0.324, *p*: 0.005, *R*: −0.327, *p*: 0.004, *R*: −0.290, *p*: 0.012, and *R*: −0.306, *p*: 0.008, respectively). However, separately investigating those cohorts indicates that the slope of the line of best fit is negative and far higher for control compared to BAV (Figure 6).

**FIGURE 7:**
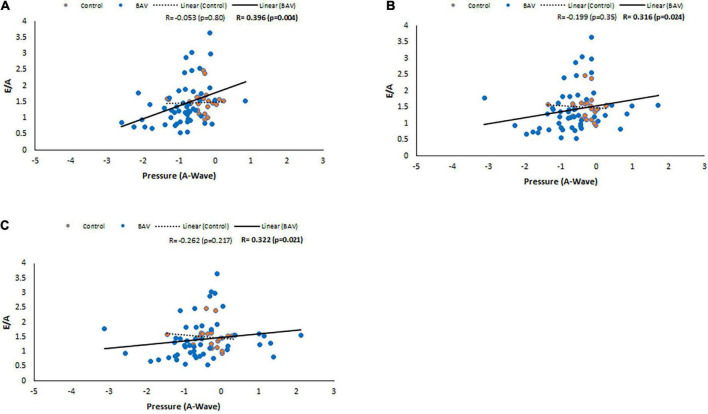
E/A correlations with pressure at A-wave timepoint in control and BAV groups. The locations are shown at **(A)** MV, **(B)** LV middle part, and **(C)** LV apex. Left atrium (LA), mitral valve (MV), and left ventricle (LV).

Following the same approach, at the locations of MV, LV middle part and LV apex, positive and statistically significant correlations were observed between E/A and pressure at A-wave timepoint (*R*: 0.347, *p*: 0.002, *R*: 0.257, *p*: 0.026, *R*: 0.253, *p*: 0.028, respectively). Note that the slope of the line of best fit is positive and higher for BAV compared to healthy controls (Figure 7). These correlations at the rest of the timepoints of the planes were negligible.

A-wave peak velocity correlates with pressure at MV plane at A-wave timepoint (*R*: −0.390, *p*: <0.001), and E-wave peak velocity has a low association with pressure at LV middle part and LV apex planes with maximum pressure (*R*: 0.365, *p*: 0.001 and *R*: 0.344, *p*: 0.003, respectively).

#### Diastolic function and age

**FIGURE 8:**
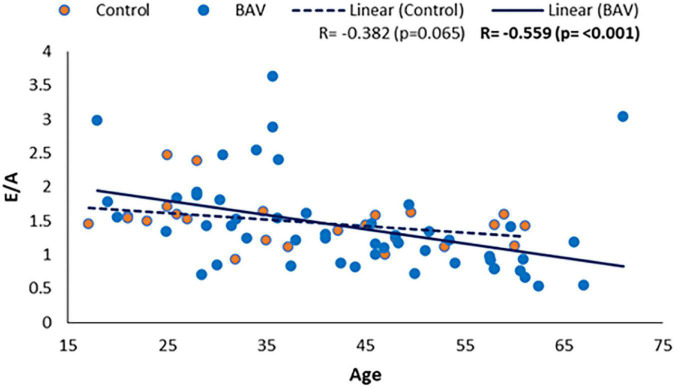
E/A correlation with age in healthy and BAV groups. BAV has a statistically significant correlation with age rather than with healthy individuals.

We also could find a moderate and negative correlation between E/A and age in our BAV cohorts (*R*: −0.559, *p*: <0.001), while it was not statistically significant between E/A and healthy control (*R* = −0.382, *p* = 0.065), indicating E/A degrade faster by aging in our BAV subjects compare to healthy individuals (Figure 8). The correlation of all subjects including healthy and BAV is statistically significant (*R* = −0.522, *p*: <0.001) as well. Therefore, the negative and statistically significant correlation between E/A and all subjects (*R*: −0.522, *p*: <0.001) is because of the BAV cohort, and the healthy cohort mitigates the negative slope of the line of best fit.

## Discussion

This study applied the 4D-flow MRI to differentiate healthy condition and BAV cohorts with mild regurgitation in terms of intraventricular flow components and pressure analysis. We also used the 4D-flow MRI capabilities to assess diastolic function in our population. Our finding indicates that while there are some degrees of LV diastolic dysfunction in the BAV cohorts with mild regurgitation and pEF, flow components as global parameters are not sensitive enough to differentiate it against the healthy condition. Therefore, the first part of our hypothesis is rejected. On the other hand, the pressure analysis at the assigned planes can reveal some local pressure differences at some timepoints, confirming our hypothesis’s second part.

Although the role of each separate component of LV flow in different cardiac diseases and remodeled LV has been assessed by application of medical imaging techniques (12, 40), we could not verify the significance of flow components as a reliable metric in asymptomatic BAV patients’ concomitant mild regurgitation and pEF since both flow components and EF are global measurements. In the correlation analysis, we could not find any considerable association between not only flow components with age, which is in alignment with Kim et al. (12) study, but also diastolic function variable.

Some published literatures emphasize the continuous impairment of AV dysfunction on the intraventricular optimum hemodynamic and structure, leading to pumping deficiency (41, 42). Hence, we targeted pressure drop as a previously validated local parameter measuring by 4D flow MRI (40–43). Pressure drop can be an early subclinical marker of diastolic dysfunction and LV remodeling in the BAV patients with mild regurgitation and pEF to provide additive values in monitoring and predicting long-term outcomes. Reproducible, non-invasive, and direct measurement of ventricular hemodynamic forces which is closely related to intracardiac pressure variations, by using 4D flow MRI in a 3-dimensional volume is also feasible with sufficient spatial and temporal resolution (44). The LV hemodynamic filling forces follow heterogeneous patterns in unhealthy subjects, and the change in magnitude of forces at early and late filling phases is linked to diastolic dysfunction (45).

Diastolic dysfunction can be classified into three subgroups according to echocardiographic indices of LV functional parameters. However, left ventricular diastolic compliance and function is a lumped parameter and interpretation of imaging indices of diastolic function is challenging and need a comprehensive knowledge of transmitral filling physiology (46).

Precise pressure gradient exchange in the healthy condition between LV and LA in the relaxation phase of LV leads to MV opening. Consequently, on-time starting and finishing of the rapid filling of the LV chamber will occur. Any deviation from the standard, optimized pressure exchange and equilibrium in the presence of morphological and hemodynamic abnormalities can lead to diastolic dysfunction. Interestingly, although there was no systolic dysfunction, we could observe diastolic dysfunction in BAV with mild regurgitation according to acquired flow and peak velocity ratio during the rapid filling pressure (RFP) (E-wave) and atrial systole (A-wave) achieved by 4D flow MRI analysis. LV filling pressure valuation has been proved reliable for evaluating HF patients with pEF (39). Optimized relaxation and compliance features of LV certify a normal stroke volume (SV) in healthy conditions, while SV means were different between control and BAV with mild regurgitation and pEF (*p*: 0.04). CO was preserved in our study cohorts, indicating that diastolic dysfunction may be developed as a compensatory mechanism by achieving some degrees of correlations between LV and LA function parameters and pressure at some planes and some timepoints.

Bicuspid aortic valve with mild regurgitation and pEF may result in diastolic dysfunction uncovering by cardiac magnetic resonance feature tracking (CMR-FT) strain imaging as a Supplementary method. Depending on the regurgitation jet direction, a significant reduction in the circumferential and especially longitudinal strain rate has been observed, indicating that they might silently be in the progressive process that finally results in HF (47).

Whilst we observed different degrees of diastolic impairment based upon the recommendations of the updated American Society of Echocardiography and European Association of Cardiovascular Imaging (ASE, EACVI) in our all-included age groups of BAV population, this phenomenon can be a normal process of aging (48). Note that LV filling pressure as a fundamental mechanism of diastolic dysfunction can be normal at the early stages of diastolic dysfunction development at rest, same as our population condition, while LV filling pressure can increase during physical activity.

Considering that BAV is accompanied by different secondary complications including LV afterload, AV defects, dilatation and dissection of thoracic aorta, local pressure drop and any clue indicating diastolic dysfunction could be the initial clinical alerts to hinder the progressive process and consequent HF with pEF.

4D flow MRI studies increasingly demonstrate the potential of this imaging technique, facilitating quantification and visualization of complex intracardiac blood flow in three orthogonal directions over time. Thus, 4D flow MRI can provide valuable and additional indices in the cases of AV abnormalities and their effects on the heart cavities, which are still poorly uncovered (25, 31, 49).

This exploratory study can provide a new insight by applying 4D flow MRI capabilities for further studies to uncover early and reliable medical imaging markers in asymptomatic and silent BAV patients with regurgitation to prognose disease development, hamper serious clinical outcomes and cardiac failure.

### Study limitation

Bicuspid aortic valve phenotypes and regurgitation jet direction were not classified in our groups with a wide range of age, which is beyond the scope of this study. In some cases, the same healthy control matched several BAV subjects. Thus, this study did not use 1:1 matching. A propensity score matching with 1:1 population ratio could be an alternative strategy for subject matching. Echocardiography and CMR-FT technique could be applied to supplement diastolic dysfunction measurement. Because of the data limitation, we did not follow up the BAV cohorts to assess any progression in terms of left ventricular remodeling, functional degradation, and surgery referral. Since this exploratory study aimed to prove the validity or accuracy of local and global measurements, all the limitations will be considered in future studies to verify the role of local pressure and its associated indices in clinical applications for disease development.

## Conclusion

In conclusion, the findings of this study propose that pressure (local measurement) can be a promising imaging biomarker compared to flow components to prognose left ventricular dysfunction in the BAV-induced regurgitation cases with pEF that may stay clinically asymptomatic for years. The role of local pressure by applying 4D flow MRI deserves more scrutiny in future studies to be validated as an early and reliable subclinical biomarker of LV diastolic dysfunction and remodeling in asymptomatic BAV patients in clinical practice for better risk stratification and disease management.

## Data availability statement

The raw data supporting the conclusions of this article will be made available by the authors, without undue reservation.

## Ethics statement

The studies involving human participants were reviewed and approved by University of Calgary Conjoint Health Research Ethics Board. The patients/participants provided their written informed consent to participate in this study.

## Author contributions

SA and JG performed the data analysis and data collection, drafted the initial manuscript, and performed and reviewed statistical analysis. AS developed the 4D-flow prototype module and reviewed data outputs. MFB, MSB, CL, PF, and JW monitored the patients, supervised the cardiac scan exam, and supervised and performed the clinical reading. JG designed the study, supervised the students and data analysis, and drafted manuscript. All authors contributed to manuscript revision, read, and approved the submitted version.
