# Supplementary material for: Intra-cardiac pressure drop and flow distribution of bicuspid aortic valve disease in preserved ejection fraction
Source: Front Cardiovasc Med. 2022 Aug 24;9:903277. doi: 10.3389/fcvm.2022.903277 (PMC9448951; doi:10.3389/fcvm.2022.903277)

Supplementary Figure.1. Bland-Altman plots. Bland-Altman test with upper and lower 95% confidence interval limits for the average difference. As an example, it has been provided for the mitral valve plane at four cardiac timepoints.

Mitral valve (MV)

Supplementary Figure 2. Color-coded pressure pattern of the LA and LV throughout the four phases of a cardiac cycle (peak systole, end-systole, E-wave and A-wave). Both subjects are age-matched and have approximately the same flow with pEF. A) shows a 53-year-old healthy female and E/A : 1.12. B) shows a 54-year-old BAV patient, male with mild regurgitation and E/A: 0.88. The BAV subject shows the different local pressure patterns compare to healthy control.

Left atrium (LA), left ventricle (LV), preserved ejection fraction (pEF), and mitral valve (MV), Mid (middle part)

SUPPLEMENTARY TABLE 1

Supplementary Table.1. Primary pressure measurements

Group	Case ID	Left Atrium Mid						Mitral Valve						Left Ventricle Mid						Left Ventricle Apex					
		Max	Avg.	Peak Sys.	End Sys.	E-wave	A-wave	Max	Avg.	Peak Sys.	End Sys.	E-wave	A-wave	Max	Avg.	Peak Sys.	End Sys.	E-wave	A-wave	Max	Avg.	Peak Sys.	End Sys.	E-wave	A-wave
0	7379	0.949	-0.084	0.575	0.128	-0.235	-0.07	1.176	0.085	1.176	0.274	-0.081	-0.064	1.062	0.202	1.062	0.36	0.232	0.04	0.945	0.234	0.896	0.409	0.378	0.024
0	7848	0.523	-0.055	-0.249	0.202	-0.476	0.148	1.129	0.227	1.129	0.623	-0.684	0.113	1.271	0.425	1.271	0.736	-0.118	0.279	1.223	0.583	1.223	0.891	0.457	0.324
0	7907	1.407	0.129	0.487	0.276	-1.407	0.208	1.784	0.278	0.392	0.69	-1.784	0.234	1.354	0.396	-0.373	0.977	-0.985	0.267	1.417	0.455	-0.809	1.092	-0.857	0.271
0	7859	3.329	0.847	1.647	0.599	0.252	-0.29	4.4	1.138	2.629	1.13	0.591	-0.71	5.798	1.102	1.682	1.144	1.066	-0.629	6.099	1.144	1.481	1.136	1.001	-0.574
0	7860	0.722	0.029	0.175	0.109	-0.527	-0.042	1.724	0.1	0.778	0.48	-1.288	-0.236	1.8	0.307	1.524	0.683	-1.22	-0.147	1.67	0.348	1.42	0.86	-1.181	-0.278
0	7904	0.319	0.004	0.025	-0.013	-0.319	-0.068	1.7	0.112	0.462	0.388	-1.678	-0.32	1.387	0.172	0.422	0.487	-1.326	-0.323	1.388	0.289	-0.107	0.619	-0.613	-0.399
0	7851	0.49	0.145	0.176	0.354	-0.273	-0.141	1.153	0.317	0.761	0.814	-0.418	-0.315	1.374	0.357	0.977	0.961	-0.055	-0.325	1.432	0.317	0.953	1.038	0.113	-0.37
0	7852	1.108	0.44	0.792	0.552	-0.408	-0.398	2.991	0.929	1.117	2.862	-1.795	-0.188	3.251	1.05	0.947	3.155	-1.239	-0.161	3.414	1.202	0.728	3.38	-0.966	0.177
0	9637	1.295	-0.49	0.512	0.257	-0.77	-0.04	2.825	-0.69	1.417	0.719	-2.189	-0.292	2.994	-0.057	1.919	1.041	-2.194	-0.145	3.278	-0.141	1.464	1.151	-2.005	-0.146
0	8494	0.422	0.04	0.422	0.186	-0.043	0.028	1.081	0.159	1.081	0.733	-0.369	-0.28	0.714	0.204	0.606	0.714	0.119	-0.024	0.723	0.188	0.133	0.723	0.264	0.013
0	1149	0.67	-0.058	0.241	0.224	-0.638	-0.117	1.533	0.046	1.157	0.591	-1.02	-0.462	1.208	0.117	1.208	0.689	-0.524	-0.264	1.162	0.175	1.162	0.705	-0.095	-0.275
0	7849	0.71	0.099	0.71	0.12	-0.359	-0.216	1.691	0.207	1.691	0.231	-0.954	-0.739	2.586	0.467	2.586	0.367	-0.387	-0.656	2.726	0.664	2.726	0.375	0.315	-0.754
0	9638	0.317	-0.062	-0.036	0.175	-0.255	-0.232	0.505	-0.055	0.362	0.391	-0.334	-0.448	0.846	-0.028	-0.13	0.524	0.188	-0.283	1.077	-0.04	-0.346	0.56	0.26	-0.244
0	1212	0.271	-0.076	-0.189	-0.245	-0.017	-0.06	0.785	-0.057	0.168	0.075	-0.188	-0.198	0.847	-0.051	0.087	0.126	-0.043	-0.041	0.549	-0.016	-0.091	0.183	0.055	-0.091
0	1223	2.128	0.425	-0.064	0.741	0.182	0.052	5.512	0.917	-0.328	2.036	-0.267	-0.455	6.362	1.159	-0.96	2.485	0.121	-0.142	6.433	1.309	-1.49	2.65	0.584	-0.023
0	4063	0.787	-0.054	0.307	-0.006	-0.359	-0.431	1.898	-0.227	0.78	-0.058	-1.267	-1.295	2.769	-0.638	0.165	0.132	-0.748	-1.351	2.236	-0.738	-0.488	0.272	0.217	-1.45
0	3337	0.479	0.172	0.406	0.271	0.201	0.081	1.166	0.31	1.07	0.549	0.041	-0.197	1.422	0.407	1.374	0.581	0.469	-0.068	1.211	0.45	1.072	0.632	0.577	0.027
0	5633	0.461	-0.064	0.309	0.003	-0.166	-0.46	0.724	-0.041	0.338	-0.141	-0.035	-0.491	0.53	-0.048	-0.272	-0.306	0.308	-0.311	0.837	-0.097	-0.742	-0.383	0.424	-0.275
0	9868	0.596	0.061	0.33	0.245	-0.218	-0.103	1.313	0.056	0.848	0.58	-0.855	-0.497	1.275	0.065	1.191	0.758	-0.566	-0.423	1.136	0.014	0.703	0.857	0.091	-0.547
0	5094	0.237	-0.018	0.089	-0.212	-0.122	0.077	0.612	-0.119	0.391	-0.334	-0.486	-0.28	0.845	-0.085	0.773	-0.399	-0.283	-0.132	0.746	-0.03	0.746	-0.397	-0.013	-0.106
0	9086	0.204	0.046	0.016	0.204	0.045	-0.01	1.319	0.051	0.761	0.529	-0.806	-0.681	1.08	0.131	1.08	0.649	-0.259	-0.57	0.934	0.079	0.712	0.71	-0.137	-0.657
0	7948	0.686	-0.245	-0.106	-0.05	-0.131	-0.445	1.327	-0.456	-0.125	0.083	-0.728	-1.327	1.189	-0.349	0.207	0.14	-0.409	-0.655	1.012	-0.298	0.064	0.15	-0.12	-0.496
0	1215	1.886	0.291	1.886	0.486	1.012	-0.275	3.172	0.408	3.172	0.55	1.163	-0.806	1.929	0.412	1.814	0.4678	1.526	-0.404	1.675	0.388	0.996	0.475	1.675	-0.287
0	1150	0.208	0.029	0.172	0.021	-0.208	0.104	1.154	0.027	1.154	0.037	-0.662	0.03	1.417	0.125	1.417	0.003	0.097	-0.004	1.346	0.168	1.346	0.011	0.227	-0.057
1	8918	0.522	0.001	-0.42	0.02	-0.163	-0.001	3.454	0.094	1.618	0.327	-0.851	-0.117	5.314	-0.009	2.419	0.367	-1.165	-0.143	5.399	0.004	2.522	0.375	-0.506	-0.177
1	3155	1.647	-0.128	-0.3	0.799	-0.155	-0.122	4.719	-0.456	-0.084	2.414	-0.869	-2.127	5.723	-0.555	-0.077	2.69	0.735	-3.114	5.346	-0.561	-0.267	2.725	0.814	-3.117
1	7175	1.464	-0.303	0.982	-1.17	-0.764	0.06	3.597	-0.423	3.597	-2.407	-2.28	-0.003	4.133	-0.525	1.861	-1.477	-2.35	0.415	4.629	-0.685	1.093	-1.305	-2.084	0.359
1	0540	1.69	-0.264	-0.161	0.101	-1.69	-0.421	4.217	-0.353	0.206	0.698	-4.217	-1	3.167	-0.277	-0.529	0.739	-3.167	-0.947	2.955	-0.253	-0.852	0.762	-2.955	-0.946
1	8702	0.666	-0.197	-0.088	-0.102	-0.666	-0.256	1.556	-0.447	0.154	-0.049	-1.556	-1.067	0.999	-0.255	0.387	-0.065	-0.876	-0.999	0.92	-0.207	0.311	-0.06	-0.704	-0.92
1	2905	0.548	0.021	-0.017	0.344	0.396	-0.02	1.673	0.019	0.686	0.589	-0.205	-0.14	1.357	0.202	0.847	0.697	0.263	-0.079	1.485	0.268	0.676	0.731	0.294	-0.116
1	6963	0.785	0.016	0.213	0.779	-0.778	-0.221	3.897	0.173	1.552	2.875	-3.897	-0.83	3.213	0.344	1.171	3.213	-3.132	-0.52	3.272	0.432	0.489	3.272	-2.568	-0.526
1	9099	1.108	-0.065	0.778	0.096	-0.453	-0.68	4.149	0.054	2.821	0.231	-1.142	-2.241	1.961	-0.322	1.455	0.107	-0.868	-1.614	1.546	-0.69	-0.589	-0.095	-0.245	-1.186
1	1078	0.532	-0.088	0.532	-0.044	-0.307	-0.299	1.903	-0.159	1.903	0.042	-1.456	-0.771	1.846	-0.09	1.846	0.013	-0.719	-0.758	1.484	-0.048	1.484	-0.015	-0.29	-0.692
1	7074	1.124	-0.015	0.364	0.477	0.225	-1.124	2.59	-0.707	-1.154	-0.022	0.243	-2.59	2.788	0.789	1.455	2.591	2.401	-1.59	3.209	1.058	1.634	2.952	2.67	-1.231
1	9181	10.93	-2.005	-10.93	1.045	-0.613	-0.298	3.881	0.547	0.994	3.881	0.319	-0.596	6.288	0.861	4.142	4.957	-0.139	-0.749	7.11	1.283	4.802	5.334	0.812	-0.681
1	8710	0.727	0.09	0.727	0.13	-0.172	-0.032	2.908	0.266	2.908	1.189	-2.19	-0.636	3.483	0.513	3.483	1.352	-1.274	-0.556	3.656	0.548	3.618	1.433	-0.73	-0.703
1	9347	0.45	0.062	0.382	0.063	-0.113	-0.056	1.927	0.029	1.927	0.198	-1.336	-0.718	1.552	-0.203	1.016	0.111	-0.578	-0.612	1.542	-0.133	0.627	0.078	-0.051	-0.518
1	4426	0.995	-0.39	-0.127	-0.995	-0.602	-0.087	2.929	-0.814	-0.114	-1.287	-2.136	-0.43	1.437	-0.55	-0.085	-1.38	-1.025	-0.137	1.567	-0.435	-0.339	-1.412	-0.704	0.03

1	8801	0.867	0.23	0.867	0.212	0.141	0.378	1.398	0.335	0.582	0.289	0.671	0.851	1.929	0.497	0.142	0.299	1.7	0.995	2.083	0.555	-0.011	0.307	1.887	1.138
1	7065	2.072	-0.859	-0.604	0.123	-2.029	-1.572	2.088	-0.548	0.587	-0.11	-1.991	-1.201	1.489	0.492	0.496	-0.541	1.059	0.245	3.302	0.995	-0.365	-0.653	2.904	1.021
1	8578	0.73	0.033	0.73	-0.322	-0.647	-0.126	1.166	-0.003	-0.08	0.071	-1.166	-0.151	1.228	0.04	-1.194	0.226	-0.488	-0.135	1.408	0.06	-1.406	0.236	-0.549	-0.115
1	7725	1.269	-0.083	1.269	0.196	-0.339	-0.272	3.009	-0.22	3.009	0.518	-1.255	-0.801	2.539	-0.162	2.539	0.484	-0.529	-0.587	1.593	0.067	1.593	0.422	-0.18	-0.314
1	5073	1.066	-0.391	-0.286	-0.162	-0.909	0.073	2.455	-0.687	-0.066	0.651	-2.31	-0.418	3.557	1.227	0.491	0.967	0.988	1.716	4.969	1.599	0.266	1.01	1.901	2.121
1	6748	0.824	-0.02	-0.383	-0.024	-0.824	0.069	4.011	-0.369	-1.786	-0.531	-4.011	-0.847	3.219	0.335	0.513	0.5	-3.219	-0.894	2.57	0.56	0.987	1.005	-2.57	-1.103
1	7071	0.222	-0.066	0.095	0.039	-0.097	-0.152	0.793	-0.187	0.418	0.107	-0.592	-0.279	0.697	0.123	0.697	0.142	0.226	-0.415	0.737	0.226	0.42	0.191	0.693	-0.571
1	1623	1.086	0.011	1.002	0.101	-0.056	-0.187	4.176	-0.066	4.176	0.968	-1.617	-1.275	4.225	-0.471	3.382	1.098	-1.878	-1.057	4.808	-0.482	2.611	1.063	-1.198	0.999
1	8605	0.901	-0.15	0.319	-0.901	-0.089	-0.028	2.069	-0.258	1.569	-1.066	-0.723	-1.001	1.955	-0.244	1.947	-1.257	-0.553	-1.017	1.426	-0.205	1.272	-1.426	-0.302	-0.85
1	1802	0.503	-0.071	-0.003	-0.09	-0.46	0.067	1.776	-0.203	0.107	0.089	-1.286	-0.539	3.124	-0.457	0.002	0.117	-1.516	-0.495	2.484	-0.438	-0.286	0.144	-1.042	-0.549
1	4506	0.468	-0.199	0.032	-0.157	-0.372	-0.415	1.49	-0.628	0.813	-0.409	-1.227	-1.411	1.38	-0.54	0.78	-0.417	-0.879	-1.353	1.271	-0.321	0.43	-0.484	0.078	-1.249
1	8060	0.297	0.01	0.25	-0.111	0.091	-0.017	2.166	-0.027	2.166	-0.414	-0.009	-0.957	2.067	-0.26	0.874	-0.639	0.786	-0.985	2.542	-0.394	-0.167	-0.767	1.132	-1.183
1	9328	1.268	0.098	0.702	0.079	-0.511	-0.261	3.101	0.745	1.691	0.808	-0.208	-0.079	2.614	0.845	1.92	1.043	0.426	0.674	2.291	0.939	1.766	1.06	0.851	1.394
1	7711	0.644	0.041	0.409	0.177	-0.062	-0.287	1.658	-0.063	1.301	0.255	-0.614	-1.219	1.573	-0.029	1.573	0.161	-0.178	-1.051	1.101	-0.029	1.008	0.013	0.112	-0.722
1	8947	0.355	0.045	0.202	0.19	-0.125	0.053	2.65	0.314	2.268	1.554	-1.827	-0.88	3.18	0.211	0.464	1.852	-1.421	-1.214	3.269	0.247	-0.035	1.887	-1.256	-1.212
1	9479	0.368	-0.011	0.338	-0.178	0.131	-0.221	0.881	-0.239	0.732	-0.646	-0.167	-0.816	0.772	-0.144	-0.082	-0.06	0.143	-0.772	1.258	-0.105	-0.846	0.309	0.445	-0.958
1	0686	0.973	0.118	0.973	0.123	-0.359	-0.011	1.128	-0.124	0.903	0.227	-1.023	-0.773	1.428	-0.355	0.77	0.369	0.479	-0.428	2.239	-0.433	-1.647	0.451	-0.189	-0.309
1	1455	0.59	0.073	0.59	-0.293	-0.116	-0.185	2.754	0.363	2.272	-0.733	-0.345	-1.132	3.155	0.223	0.96	-0.702	0.692	-0.728	3.181	0.109	0.015	-0.709	0.963	-0.725
1	9435	0.993	-0.269	0.415	0.055	-0.464	-0.294	3.515	-0.789	1.148	0.07	-2.554	-0.603	0.969	0.288	0.582	-0.169	0.128	0.851	1.746	0.617	-0.016	-0.278	1.302	1.304
1	7411	0.412	0.002	0.409	0.1	-0.081	-0.075	0.976	-0.08	0.241	0.362	-0.697	-0.385	0.592	-0.139	-0.47	0.491	-0.111	-0.274	0.803	-0.094	-0.757	0.517	0.186	-0.261
1	6494	1.872	0.02	1.872	-0.403	-0.723	-0.374	5.298	0.451	5.298	0.28	-1.734	-1.943	2.63	0.131	1.934	0.58	-2.439	-1.766	2.133	-0.128	-1.04	0.937	-1.706	-1.668
1	9232	1.451	-0.201	0.237	0.228	-0.7	0.011	3.135	-0.45	1.742	0.253	-1.965	-0.783	3.168	-0.535	1.352	0.45	-1.895	-0.781	3.691	-0.561	0.56	0.59	-1.774	-0.906
1	0209	0.562	-0.056	0.14	0.081	-0.094	-0.07	1.415	-0.189	0.694	0.282	-1.12	-0.379	1.792	0.182	1.501	0.649	-0.776	0.044	1.849	0.442	1.465	0.929	-0.053	0.153
1	0718	6.478	-1.095	-1.429	0.496	-2.104	-0.562	4.4	-0.804	0.364	0.315	-2.585	-0.718	2.834	-0.44	1.59	0.283	-2.38	-0.621	3.217	-0.371	1.441	0.351	-2.105	-0.977
1	1139	0.287	0.031	0.273	-0.184	-0.127	-0.138	0.996	-0.009	0.996	-0.258	-0.739	-0.757	0.993	0.051	0.993	-0.316	-0.243	-0.685	0.718	0.088	0.718	-0.326	-0.1	-0.709
1	3680	0.797	-0.013	0.221	-0.057	-0.179	-0.266	1.07	-0.14	0.705	0.304	-0.903	-1.07	2.38	0.078	1.267	0.209	-0.74	-0.939	2.498	0.241	0.978	0.257	-0.367	-0.677
1	7939	1.529	0.281	0.566	0.116	0	0.059	3.411	0.668	2.053	0.688	-0.386	-1.377	3.202	0.676	1.614	0.676	0.091	-1.328	2.959	0.683	1.121	0.708	0.265	-1.413
1	0802	2.499	0.701	1.057	0.702	0.414	-0.055	4.783	0.999	2.507	0.856	-0.334	-0.737	5.74	1.084	2.537	1.136	-0.815	-0.442	5.905	1.214	2.026	1.337	0.673	-0.82
1	8252	0.387	0.028	0.145	-0.006	0.056	-0.028	1.179	-0.032	0.296	-0.099	-0.138	-0.675	1.461	0.063	0.565	0.073	0.017	-0.447	1.19	0.109	0.366	0.335	0.122	-0.48
1	9252	7.96	0.97	7.2	0.9	-1.01	-0.9	11.93	1.43	11.9	0.9	-2.6	-1.8	9.91	1.48	9.9	0.9	-0.9	-1.2	8.54	1.48	8.3	0.9	-0.2	-1.1
1	8345	1.434	-0.381	-1.408	0.245	-0.163	-0.642	2.029	-0.556	-0.781	0.33	-0.484	-2.029	2.267	-0.262	0.748	0.337	-0.201	-2.267	2.561	-0.029	1.707	0.458	0.139	-2.561
1	8593	0.827	0.052	0.827	0.13	-0.211	0.075	6.979	0.344	6.979	1.803	-2.109	-1.748	5.879	0.297	5.879	1.976	-2.434	-1.954	4.453	-0.07	4.013	2.24	-2.689	-1.888
1	8631	1.073	0.193	0.653	0.104	-0.229	-0.353	1.777	0.328	1.407	0.221	-0.35	-0.694	1.245	0.248	1.245	0.5	0.407	-0.054	1.018	0.24	0.887	0.474	0.576	0.178
1	4665	0.461	0.12	-0.125	0.428	0.056	0.085	1.099	-0.073	-0.574	0.557	-0.094	-0.761	1.142	0.02	-0.114	0.908	-0.079	-0.989	1.248	0.284	0.029	1.153	0.304	-0.96
1	4666	0.461	-0.088	0.089	-0.047	-0.204	-0.201	1.093	-0.337	0.144	-0.092	-0.738	-0.64	0.796	-0.109	0.309	-0.036	-0.188	-0.393	0.884	-0.053	0.219	-0.025	-0.081	-0.269
1	2740	1.566	0.109	1.267	-0.089	-0.279	-0.176	5.891	0.491	5.365	0.437	-1.068	-1.105	0.723	-0.296	-0.316	-0.558	-0.356	-0.603	3.495	-0.648	-3.495	-1.06	0.238	-0.208
1	0269	0.28	0.008	-0.15	0.071	-0.01	-0.28	1.85	-0.202	-0.869	0.277	-0.311	-0.958	2.002	-0.049	0.105	0.19	-0.092	-0.554	1.987	0.004	0.292	0.165	-0.03	-0.358

Groups: Control (0), BAV (1)

SUPPLEMENTARY TABLE 2

Supplementary Table.2. Repeated pressure measurements

Case ID	Left Atrium Mid						Mitral Valve						Left Ventricle Mid						Left Ventricle Apex					
	Max	Avg.	Peak Sys.	End Sys.	E-wave	A-wave	Max	Avg.	Peak Sys.	End Sys.	E-wave	A-wave	Max	Avg.	Peak Sys.	End Sys.	E-wave	A-wave	Max	Avg.	Peak Sys.	End Sys.	E-wave	A-wave
7848	0.518	-0.209	-0.031	-0.192	-0.412	-0.237	1.166	-0.33	-0.165	0.026	-1.043	-0.368	1.434	-0.14	0.587	0.49	-0.79	-0.412	1.352	0.026	0.426	0.679	0.075	-0.414
7851	0.491	0.132	0.468	0.237	-0.047	-0.115	1.452	0.341	1.452	0.846	-0.545	-0.524	1.584	0.362	1.497	1.043	0.256	-0.54	1.616	0.317	1.396	1.08	0.486	-0.576
9637	1.632	-0.098	0.487	0.419	-1.132	-0.085	2.81	-0.09	1.301	0.955	-2.247	-0.061	2.302	0.026	1.47	1.159	-1.66	-0.0001	2.18	0.089	1.281	1.228	-1.207	0.01
9638	0.305	0.003	0.08	0.239	-0.12	-0.305	0.462	-0.034	0.131	0.371	-0.272	-0.462	0.585	0.12	0.258	0.585	0.05	-0.327	0.746	0.106	-0.191	0.677	0.12	-0.285
9086	0.6	0.141	0.6	0.458	0.093	-0.268	1.268	0.111	1.268	0.727	-0.798	-0.722	1.693	0.193	1.693	0.832	-0.462	-0.67	1.387	0.153	1.387	0.9	-0.461	-0.782
7175	1.241	-0.028	0.935	-0.763	-1.068	0.514	2.201	-0.201	0.954	-0.718	-2.174	0.667	2.17	-0.603	-1.059	-0.348	-1.668	1.145	2.302	-0.746	-1.952	-0.271	-1.027	1.224
0540	1.487	-0.227	-0.04	-0.089	-1.487	-0.457	3.073	-0.385	-0.115	0.416	-3.073	-0.988	2.28	-0.379	-0.928	0.422	-2.28	-1.079	2.259	-0.376	-1.017	0.429	-2.229	-1.084
7074	1.53	0.326	1.53	1.056	0.072	-1.063	2.181	0.397	2.181	1.59	0.319	-1.828	1.998	0.362	0.717	1.418	1.304	-1.459	2.533	0.349	-0.64	1.366	1.895	-0.984
5073	1.928	-0.463	0.307	0.374	-0.971	-1.235	1.181	-0.133	0.353	0.785	-1.121	-0.567	4.316	1.598	1.26	1.091	1.983	1.276	5.189	1.802	1.198	1.11	2.402	1.512
1623	0.952	0.082	0.952	0.152	0.001	-0.117	3.195	0.035	3.195	0.687	-1.283	-1.133	3.217	-0.126	3.217	0.749	-1.307	-1.094	2.819	-0.17	2.819	0.683	-1.158	-1.129
9435	0.872	-0.094	0.565	0.109	-0.239	-0.272	2.693	-0.344	1.283	0.172	-1.733	-0.378	1.264	-0.015	0.96	-0.09	-0.839	0.346	1.029	0.086	0.451	-0.196	-0.115	0.581
8345	1.041	0.039	1.032	-0.04	-0.287	-0.466	1.126	-0.21	0.761	-0.034	-0.54	-1.11	2.079	-0.387	-1.582	0.152	0.043	-0.514	3.955	-0.484	-3.047	0.3	0.448	-0.324
8593	0.825	0.146	0.825	0.511	-0.236	0.045	3.334	0.391	3.334	1.158	-0.79	-0.888	3.338	0.224	3.338	1.477	-1.407	-1.76	3.007	0.042	2.389	1.875	-2.085	-1.962
8631	1.454	0.082	-0.095	0.197	-0.244	-0.245	3.058	0.363	2.064	0.772	-0.822	-1.414	2.906	0.152	1.943	0.657	-0.596	-1.325	1.91	-0.001	0.74	0.482	-0.23	-1.036
9099	1.028	-0.119	0.28	0.18	-0.68	-0.802	3.369	-0.06	1.76	0.36	-1.536	-2.206	1.419	-0.311	0.28	0.19	-0.421	-1.419	1.325	-0.461	-0.561	0.052	0.025	-1.069
7379	0.822	0.204	0.753	0.254	0.313	0.005	1.354	0.349	1.219	0.411	0.711	-0.025	1.268	0.489	1.152	0.517	1.222	0.119	1.436	0.458	0.66	0.607	1.372	0.093
7852	0.704	0.024	-0.063	0.635	-0.121	0.119	3.422	0.661	-0.018	3.422	-0.93	0.842	3.533	0.76	-0.165	3.533	-0.189	1.016	3.674	0.949	-0.418	3.674	0.506	1.382
8494	0.202	0.073	0.19	0.184	-0.035	-0.061	0.554	0.107	0.554	0.336	-0.248	-0.373	0.516	0.191	0.322	0.347	0.306	-0.211	0.657	0.191	-0.032	0.338	0.487	-0.193
7948	0.279	-0.127	-0.0001	-0.107	-0.198	-0.068	1.361	-0.468	-0.149	-0.107	-1.199	-1.149	1.182	-0.349	0.416	-0.03	-0.914	-0.542	0.909	-0.307	0.383	-0.016	-0.669	-0.477
1139	0.533	0.051	0.395	0.033	-0.166	-0.384	1.853	0.138	1.454	0.192	-0.687	-1.035	1.163	0.058	1.075	0.093	-0.299	-0.841	0.803	-0.012	0.239	-0.008	0.044	-0.803
3155	1.87	-0.261	-0.01	0.854	-0.97	-0.571	3.434	-0.39	0.233	1.562	-2.268	-0.89	3.953	-0.393	0.012	1.688	-1.002	-1.053	2.35	-0.191	-0.374	1.773	-0.069	-0.586
1078	0.395	0.004	0.263	0.027	0.074	-0.045	1.939	-0.054	1.444	0.056	-0.822	-0.665	1.747	-0.076	1.354	-0.052	-0.172	-0.854	1.476	-0.059	1.103	-0.093	0.142	-0.984
1802	0.419	0.013	0.315	-0.014	-0.119	-0.138	1.112	-0.276	0.019	-0.053	-1.024	-0.695	1.371	-0.37	-0.635	-0.081	-0.109	-0.32	1.343	-0.282	-1.067	-0.084	0.614	-0.231
9479	0.415	-0.067	-0.004	-0.077	0.023	-0.109	0.853	-0.165	0.499	-0.326	-0.059	-0.853	1.37	0.472	0.988	0.807	0.655	-1.091	1.948	0.672	0.817	1.156	0.99	-1.078
0686	0.576	-0.147	0.244	-0.064	-0.576	-0.285	0.607	-0.233	-0.255	-0.049	-0.536	-0.555	1.473	-0.107	-1.275	0.006	0.24	0.163	2.1	-0.046	-1.929	0.038	0.768	0.405

SUPPLEMENTARY FIGURE 1

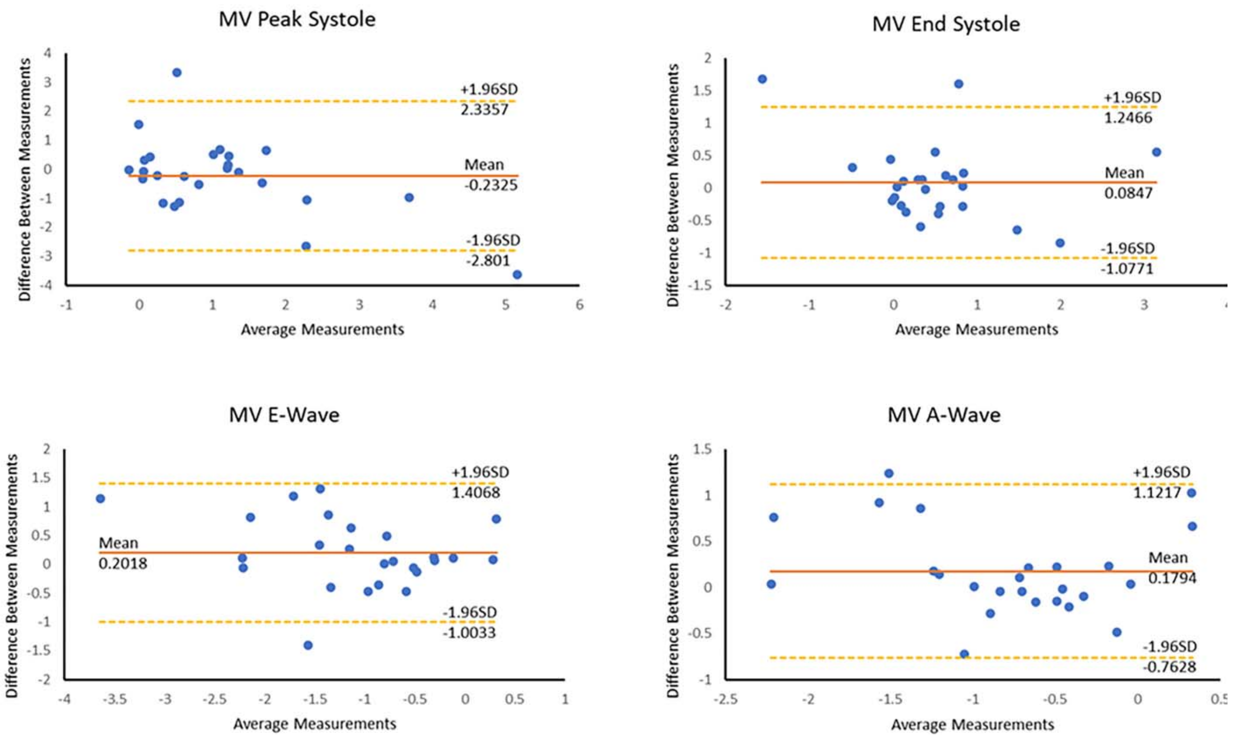

Supplementary Table.3. Bias and limits of agreements from Bland-Altman plots analysis

**Plains**		**-1.96 SD**	**Mean**	**+1.96 SD**
Left Atrium Mid	Max	-0.6084	-0.0020	0.6044
	Avg	-0.3334	0.0530	0.4395
	Peak Systole	-1.1753	0.1159	1.4070
	End Systole	-0.3663	0.0865	0.5393
	E-wave	-0.5458	0.0002	0.5462
	A-wave	-0.7464	-0.0507	0.6450
Mitral Valve	Max	-2.3124	-0.4534	1.4056
	Avg	-0.5050	0.1199	0.7448
	Peak Systole	-2.8007	-0.2325	2.3357
	End Systole	-1.0771	0.0847	1.2466
	E-wave	-1.0033	0.2018	1.4068
	A-wave	-0.7628	0.1794	1.1217
Left Ventricle Mid	Max	-2.1792	-0.3079	1.5634
	Avg	-0.4856	0.0150	0.5155
	Peak Systole	-2.5472	-0.4426	1.6620
	End Systole	-1.0001	-0.0443	0.9116
	E-wave	-1.4161	0.1510	1.7180
	A-wave	-1.2681	0.1260	1.5202
Left Ventricle Apex	Max	-2.3697	-0.3662	1.6372
	Avg	-0.6505	0.0069	0.6643
	Peak Systole	-3.0627	-0.4670	2.1286
	End Systole	-1.0734	-0.0721	0.9292
	E-wave	-1.2546	0.2330	1.7206
	A-wave	-1.8005	0.0708	1.9421

Max stand for maximum; Mid, middle part; Avg, average; SD, standard deviation

SUPPLEMENTARY FIGURE 2

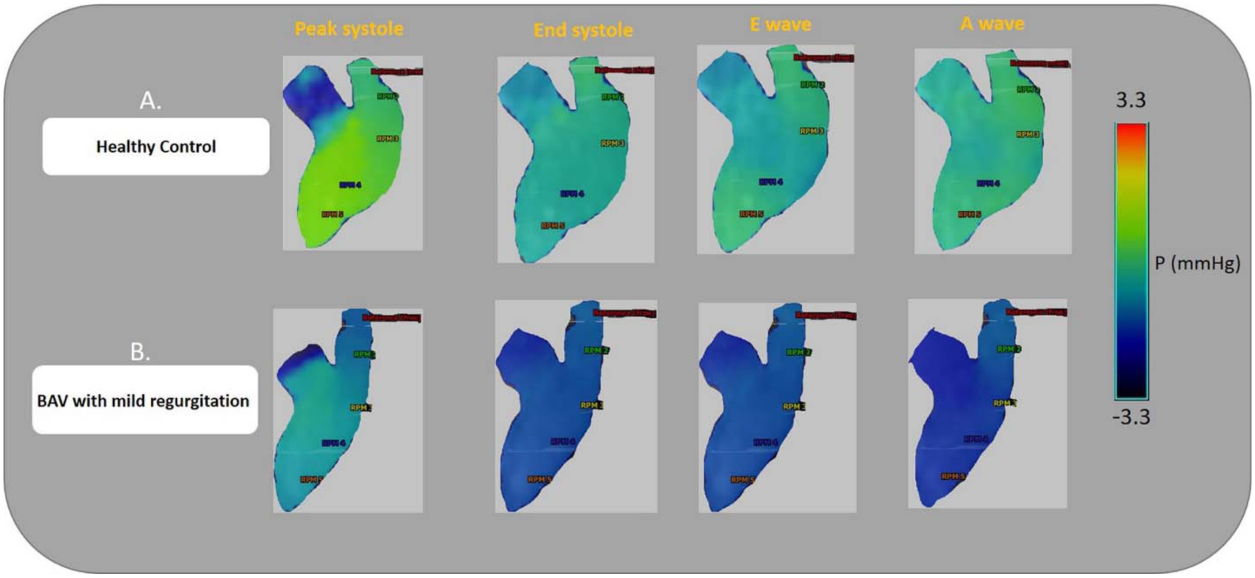

Supplement: Supplementary file 1 [file Data_Sheet_1.pdf]
